# GUIDELINES FOR DEVELOPING EVIDENCE-BASED RISKY DRIVING COUNTERMEASURES THAT INCLUDE OLDER DRIVERS

**DOI:** 10.1093/geroni/igac059.655

**Published:** 2022-12-20

**Authors:** Jennifer Zakrajsek, Lisa Molnar, David Eby, Lidia Kostyniuk, Nicole Zanier, David J LeBlanc, Tina B Sayer

**Affiliations:** University of Michigan Transportation Research Institute, Ann Arbor, Michigan, United States; University of Michigan Transportation Research Institute, Ann Arbor, Michigan, United States; University of Michigan Transportation Research Institute, Ann Arbor, Michigan, United States; University of Michigan Transportation Research Institute, Ann Arbor, Michigan, United States; University of Michigan Transportation Research Institute, Ann Arbor, Michigan, United States; University of Michigan Transportation Research Institute, Ann Arbor, Michigan, United States; Toyota Motor North America Research & Development, Ann Arbor, Michigan, United States

## Abstract

Driver behavior will continue to play a critical role in driving safety for the foreseeable future. Utilizing behavior change theory appropriately presents opportunities to improve the effectiveness of risky driving countermeasures that have been under-utilized to date. Older drivers should not be excluded from consideration of risky behaviors. Forty-six drivers (33% age 65+) completed surveys, then drove for three weeks with data collection during all trips. The Theory of Planned Behavior guided a two-phased regression analysis approach: 1) behavioral intentions were predicted using attitudes about behaviors and demographics; 2) observed risky behavior was predicted using behavioral intentions, theory constructs, personality/psychosocial characteristics, demographics, and driving exposure. Results were synthesized and the emergent themes were used to formulate guidelines for developing theory-based education and communication risky driving countermeasures. Guidelines focused on four risky driving behaviors observed in a large proportion of participants (72% - 96%): holding/using a cellphone; eating/drinking; speeding; and tailgating. Twenty-six guidelines were developed across four categories: 1) relationships among risky behaviors; 2) characteristics or underlying dimensions of risky driving (e.g., time, location, emotion); 3) behavior change theory constructs; 4) audience and message factors. While older drivers self-reported low frequencies of risky behaviors, low intentions for future risky behaviors, and less favorable attitudes toward risky behaviors than younger drivers they were regularly observed engaging in risky behaviors: distracted behaviors in 79% of trips and 2.1 speeding events per trip. Risky driving countermeasures are appropriate for older drivers and the emergent guidelines will be presented with recommended variations for older drivers.

